# The rs1256328 (*ALPL*) and rs12654812 (*RGS14*) Polymorphisms are Associated with Susceptibility to Calcium Nephrolithiasis in a Taiwanese population

**DOI:** 10.1038/s41598-019-53261-8

**Published:** 2019-11-21

**Authors:** Wei-Chiao Chen, Wan-Hsuan Chou, Hou-Wei Chu, Chi-Chen Huang, Xiao Liu, Wei-Pin Chang, Yii-Her Chou, Wei-Chiao Chang

**Affiliations:** 1 Institute of Clinical Pharmacy and Pharmaceutical Sciences, College of Medicine, National Cheng Kung University, Tainan, Taiwan; 2 Department of Urology, Kaohsiung Medical University Hospital, Kaohsiung, Taiwan; 3 Department of Clinical Pharmacy, School of Pharmacy, Taipei Medical University, Taipei, Taiwan; 4 Institute of Biomedical Sciences, Academia Sinica, Taipei, Taiwan; 5 Graduate Institute of Neural Regenerative Medicine, College of Medical Science and Technology/Center for Neurotrauma and Neuroregeneration, Taipei Medical University, Taipei, Taiwan; 6 Department of Human Genetics, The University of Chicago, Chicago, USA; 7 School of Health Care Administration, College of Management, Taipei Medical University, Taipei, Taiwan; 8 Master Program for Clinical Pharmacogenomics and Pharmacoproteomics, School of Pharmacy, Taipei Medical University, Taipei, Taiwan; 9 Integrative Research Center for Critical Care, Wan Fang Hospital, Taipei Medical University, Taipei, Taiwan; 10 Department of Medical Research, Shuang Ho Hospital, Taipei Medical University, New Taipei City, Taiwan

**Keywords:** Genetic predisposition to disease, Renal calculi

## Abstract

Nephrolithiasis is a common disease affecting almost all populations, with an increasing prevalence over the past decades. Previous studies revealed several functional polymorphisms associated with the pathogenesis of nephrolithiasis. However, data on Asian populations are limited. In this study, three candidate polymorphisms were selected from previous studies to investigate the correlations with nephrolithiasis in a Taiwanese population. In total, 454 nephrolithiasis patients were recruited from Kaohsiung Medical University Hospital, with SNP frequency for 1513 subjects of general population from the Taiwan Biobank (TWB) as a genotypic reference. Results revealed that subjects with minor TT genotype at rs1256328 (*alkaline phosphatase, liver/bone/kidney* (*ALPL*)) have higher susceptibility to nephrolithiasis (odds ratio (OR) = 2.03, *p* = 0.0013). In addition, subjects carrying the minor AA genotype at rs12654812 (*regulator of G protein signaling 14 (RGS14)*) have higher susceptibility to nephrolithiasis (OR = 1.91, *p* = 0.0017). Among nephrolithiasis patients, subjects with GG at rs7627468 (*calcium-sensing receptor* (*CASR*)) have lower pH level in urine (*p* = 0.0088). Importantly, rs7627468 is associated with the expressions of *IQCB1* and *EAF2.* rs12654812 could influence the expression of *RGS14* itself, *MXD3*, and *FGFR4*. In summary, this study successfully validated the genetic roles of rs1256328 and rs12654812 in human nephrolithiasis.

## Introduction

Kidney stone disease (nephrolithiasis) affects almost all populations worldwide with an increasing prevalence over the past few decades. The prevalence rate of nephrolithiasis was reported to be 5~15% in developed countries, but was as high as 20~25% in the Middle East^1^. In the United States, the prevalence of kidney stones was estimated to have increased from 3.8% in the 1970s to 8.8% in the 21st century^2,3^. In Asia, the prevalence of nephrolithiasis is 1–5%^4^.

Although many patients are persistently asymptomatic, some patients occasionally pass stones and experience hematuria. Paroxysmal renal colic may also occur and cause variable levels of pain^5,6^. Furthermore, nephrolithiasis is a disease of high recurrence risk with a relapse rate of 50% within 5~10 years and 75% at 20 years^7,8^. Continuous recurrence of kidney stone may lead to persistent renal obstruction and even permanent renal damage^6^. As a result, nephrolithiasis significantly influences patients’ quality of life and places a heavy burden on society and healthcare systems.

Many factors were suggested to elevate risks of nephrolithiasis, such as environmental exposure, low fluid intake, dietary intake, hypercalciuria, genetics, and a family’s health history. However, the etiology of nephrolithiasis remains unclear. A previous study of paired twins reported a higher concordance rate of the disease in monozygotic twins than that in dizygotic twins, suggesting that genetic factors may play a pivotal role in its etiology^9^. Furthermore, candidate gene association studies attempted to assess the roles of several genes involved in stone formation^10^. Previous studies indicated that genetic variances of the *vitamin D receptor* (*VDR*), *osteopontin* (*OPN*), and *calcium-sensing receptor* (*CASR*) genes were highly associated with nephrolithiasis^11–17^. The *TRPV5* and *CLCN5* genes were also proven to be important in the pathogenesis of nephrolithiasis^18,19^. In addition, genetic polymorphisms of calcium channel, *ORAI1* (rs12313273, rs7135617, and rs6486795), were reporetd to associate with the development of nephrolithiasis^20^ *ITPKC* (rs2607420) is also associated with the estimated creatinine clearance in nephrolithiasis patients^21^.

Recently, some regions were found to be associated with nephrolithiasis by genome-wide association studies (GWASs) or meta-analysis of GWASs in various populations^22–25^. The first GWAS on idiopathic calcium oxalate urolithiasis was published in 2009 that identified a member of the claudin gene family (*CLDN14*) as a risk locus^22^. Three loci at 5q35.3, 7p14.3, and 13q14.1 were reported to associate with kidney stones with an accumulative effect in a Japanese population^23,24^. Recently, Oddsson *et al*. indicated that four loci were associated with nephrolithiasis in an Icelandic population^25^, but this association has not been confirmed in any Asian population yet. The purpose of this study was to investigate whether the selected polymorphisms from the previous GWAS report^25^ are associated with nephrolithiasis in a Taiwanese population.

In this study, nephrolithiasis patients were recruited from Kaohsiung Medical University Hospital (KMUH, Kaohsiung, Taiwan). Genotypic statistics of general population was obtained from the Taiwan Biobank (TWB) project. We investigated the susceptibility variants with risk of nephrolithiasis in the Taiwanese population. Furthermore, associations of genetic variants with the stone frequency, stone number, clinical biochemical data, and estimated glomerular filtration rate (eGFR) were analyzed.

## Results

### Demographic and clinical characteristics of subjects

We investigated the regional prevalence of nephrolithiasis through the analysis of longitudinal health insurance data from the National Health Insurance Research Database. The prevalence of nephrolithiasis in Taiwan varies by geographical region. The prevalence of nephrolithiasis in 2010 was 3.9%, 5.95%, 4.62%, and 3.78% in northern, central, southern, and eastern Taiwan, respectively (Fig. S1). In this study, 454 nephrolithiasis patients were recruited (Table 1). Among these patients, the mean age was 53.4 ± 12.4 (SD) years, and 321 (70.7%) were male. The mean uric acid-to-creatinine ratio (UA/UCr), calcium-to-creatinine ratio (UCa/UCr), and creatinine levels in the urine were 0.5 ± 0.2, 0.1 ± 0.1, and 87.3 ± 56.3 mg/dL, respectively. As to serum data, the mean uric acid, calcium, and creatinine concentrations were 6.4 ± 1.7, 8.9 ± 0.6, 1.2 ± 0.9 mg/dL, respectively. In addition, genotypic statistics of 1,513 healthy subjects were obtained as general population from the next-generation sequencing data in Taiwan Biobank project. More than eighty percent of these subjects were recruited in southern and northern Taiwan (Fig. S2). Frequency of each polymorphism in these 1,513 subjects was used as genotypic reference of general population in this study. The frequencies of three SNPs from the 1,513 subjects were in Hardy-Weinberg equilibrium (Table 2).

**Table 1:** Basal characteristics of 454 patients with nephrolithiasis and 1,513 control subjects from Taiwan Biobank.

Characteristics	Case (n = 454)	TWB^a^ (n = 1,513)
**Age (years)**	53.4 ± 12.4	49.5 ± 11.2
Range	19–86	30–70
**Gender**		
Male	321 (70.7%)	761 (50.3%)
Female	133 (29.3%)	752 (49.7%)
**BMI**	26.5 ± 4.2	24.4 ± 3.6
**Urine data**		
UUA/UCr^b^	0.5 ± 0.2	—
UCa/UCr^c^	0.1 ± 0.1	—
Creatinine (mg/dL)	87.3 ± 56.3	—
**Serum data**		
Uric acid (mg/dL)	6.4 ± 1.7	—
Calcium (mg/dL)	8.9 ± 0.6	—
Creatinine (mg/dL)	1.2 ± 0.9	—
**eGFR**		
MDRD (mL/min 1.73 m^2^)	80.2 ± 28.4	—
CG-GFR (mL/min)	84.4 ± 28.7	—

^a^General population from Taiwan Biobank.

^b^Urinary uric acid-to-creatinine ratio (UUA/UCr).

^c^Urinary calcium-to-creatinine ratio (UCa/UCr).

**Table 2:** The basic characteristics of the SNPs (ALPL, CASR, RGS14) in different populations.

Gene	position (hg38)	variant	Allele		Minor allele frequencies (MAF)					HWE
			Major	Minor	AFR	AMR	ASN	EUR	TWB	
*ALPL*	Chr1:21570274	rs1256328	C	T	0.09	0.14	0.18	0.17	0.20	0.57
*CASR*	Chr3:122227252	rs7627468	A	G	0.75	0.82	0.46	0.76	0.48	0.36
*RGS14*	Chr5:177367190	rs12654812	G	A	0.32	0.35	0.26	0.34	0.24	0.13

AFR, African. AMR, American, ASN, Asian. EUR, European. MAFs of TWB were obtained from Taiwan View website. Minor allele determined by MAF of TWB. HWE *p* value for Hardy-Weinberg equilibrium.

### SNPs rs1256328 and rs12654812 are associated with susceptibility to calcium nephrolithiasis

To investigate associations of polymorphisms with nephrolithiasis susceptibility, we compared the genotype and allele distributions between groups of nephrolithiasis patients and general populations for each SNP (Supplementary Table S1). Among three SNPs examined, rs1256328 (*ALPL*) and rs12654812 (*RGS14*) revealed significant differences in genotypic distributions, even after correction with SNP numbers (*p* = 0.0005 and 0.0072, respectively). rs12654812 (*RGS14*) also revealed a borderline significant difference in its allelic distribution (*p* = 0.0346). However, the significance did not persist after applying Bonferroni correction. For rs7627468 (*CASR*), no significant difference was observed. On the other hand, we also investigated associations of these two SNPs (rs1256328 and rs12654812) with nephrolithiasis susceptibility under different genetic models (Tables 3, 4). As to rs1256328 (*ALPL*) and rs12654812 (*RGS14*), the most significant associations were observed under a recessive model (*p* = 0.0013 and 0.0017, respectively), with estimated ORs of 2.03 and 1.91, respectively. These results indicate that subjects with the minor TT genotype at rs1256328 (*ALPL*) or the minor AA genotype at rs12654812 (*RGS14*) had higher susceptibility to nephrolithiasis, compared to those with major alleles in these two SNPs (C allele at rs1256328 (*ALPL*) or G allele at rs12654812 (*RGS14*)).

**Table 3:** Association of rs1256328 with nephrolithiasis susceptibility.

Model	Genotype	Case (%)	TWB^a^ (%)	OR (95% CI)	*P* value
Genotypic	TT	33 (7.5)	58 (3.9)	1.87 (1.19–2.92)	**0.0055**
	CT	114 (26.0)	493 (32.7)	0.76 (0.60–0.97)	0.0254
	CC	291 (66.5)	955 (63.4)	Reference	—
Dominant	TT + CT	147 (33.5)	551 (36.6)	0.88 (0.70–1.10)	0.2454
	CC	291 (66.5)	955 (63.4)	Reference	—
Recessive	TT	33 (7.5)	58 (3.9)	2.03 (1.31–3.16)	**0.0013**
	CT + CC	405 (92.5)	1448 (96.1)	Reference	—
Trend test					0.8313

^a^General population from Taiwan Biobank. *P* values which remain significant after performing Bonferroni correction (*p* ≤ 0.016) are shown in bold.

**Table 4:** Association of rs12654812 with nephrolithiasis susceptibility.

Model	Genotype	Case (%)	TWB^a^ (%)	OR (95% CI)	*P* value
Genotypic	AA	39 (8.6)	68 (4.9)	1.93 (1.27–2.93)	**0.0019**
	AG	160 (37.1)	523 (37.6)	1.03 (0.82–1.92)	0.8191
	GG	238 (54.3)	799 (57.5)	Reference	—
Dominant	AA + AG	199 (45.7)	591 (42.5)	1.13 (0.91–1.40)	0.2664
	GG	238 (54.3)	799 (57.5)	Reference	—
Recessive	AA	39 (8.6)	68 (4.9)	1.91 (1.27–2.87)	**0.0017**
	AG + GG	398 (91.4)	1322 (95.1)	Reference	—
Trend test					0.0346

^a^General population from Taiwan Biobank. *P* values which remain significant after performing Bonferroni correction (*p* ≤ 0.016) are shown in bold.

### Polymorphisms of rs1256328, rs7627468, and rs12654812 were not associated with the risk of multiple stones or recurrence in patients with calcium nephrolithiasis

In our study, 224 patients had multiple stones and 203 had a single stone. Furthermore, 188 patients had recurrent episodes and 229 patients had a single episode. We tested whether the genotypes of these three SNPs (rs1256328, rs7627468, and rs12654812) were associated with the risk of multiple stones or recurrence of nephrolithiasis. As shown in Supplementary Table S2, we found no significant associations of these SNPs with the risk of multiple stones or recurrent calcium nephrolithiasis.

### Quantitative trait locus analysis in nephrolithiasis patients

To understand relationships between polymorphisms and clinical risk factors, biochemical quantitative traits including calcium, uric acid, and creatinine in urine and in serum were analyzed. Among the urine data, we found that rs7627468 (*CASR*) was highly associated with the urine pH, before or after adjustment with gender and age (Table 5). Urine pH levels were lower in the GG genotype group in rs7627468 (*CASR*) than in the AG or AA genotype groups (*p* = 0.009; adjusted *p* = 0.0088). In addition, the rs12654812 (*RGS14*) AA genotype was potentially correlated with an increased UCa/UCr compared to the AG or GG genotypes in nephrolithiasis patients (*p* = 0.0764; adjusted *p* = 0.0532). After the subset analysis, we found that the *CASR* SNP (rs7627468) was associated with the UCa/UCr (*p* = 0.0170) (Table 6), and the estimated OR was 0.64. However, this association did not exist after applying multiple testing correction.

**Table 5:** Association analysis between SNPs and clinical biochemical data in patients with kidney stone.

SNP	Genotype	Urine Creatinine (mg/dl)	Urine pH	UCa/UCr^a^	UUA/UCr^b^	Serum Creatinine (mg/dl)	Serum Calcium (mg/dl)	Serum Uric Acid (mg/dl)
*ALPLrs1256328*	TT	71.2 ± 42.2	6.2 ± 0.9	0.2 ± 0.1	0.5 ± 0.2	1.4 ± 1.4	8.8 ± 0.5	6.2 ± 1.5
	CT	87.6 ± 54.2	6.0 ± 0.6	0.1 ± 0.1	0.5 ± 0.2	1.2 ± 1.0	8.9 ± 0.6	6.5 ± 1.6
	CC	87.9 ± 58.6	6.0 ± 0.6	0.1 ± 0.1	0.5 ± 0.2	1.1 ± 0.8	8.9 ± 0.7	6.4 ± 1.8
	*P*-value	0.3634	0.1639	0.4293	0.9179	0.3790	0.3218	0.6075
	*P*_*adj*_-value	0.3362	0.1625	0.3798	0.9100	0.3558	0.3217	0.5633
*CASRrs7627468*	GG	95.1 ± 62.9	5.8 ± 0.6	0.1 ± 0.1	0.5 ± 0.2	1.2 ± 1.0	8.9 ± 0.7	6.4 ± 1.7
	AG	90.0 ± 58.1	6.1 ± 0.7	0.1 ± 0.1	0.5 ± 0.2	1.1 ± 0.9	8.9 ± 0.6	6.4 ± 1.6
	AA	79.0 ± 49.0	6.1 ± 0.7	0.1 ± 0.1	0.5 ± 0.1	1.2 ± 0.9	9.0 ± 0.7	6.5 ± 1.9
	*P*-value	0.0921	**0.0090**	0.8526	0.8115	0.5804	0.4067	0.8801
	*P*_*adj*_-value	0.0739	**0.0088**	0.8319	0.7946	0.5616	0.4069	0.8633
*RGS14rs12654812*	AA	76.5 ± 44.2	5.9 ± 0.6	0.2 ± 0.1	0.5 ± 0.1	1.4 ± 1.4	9.0 ± 0.6	6.5 ± 2.1
	AG	90.1 ± 58.0	6.1 ± 0.6	0.1 ± 0.1	0.5 ± 0.2	1.1 ± 0.6	8.9 ± 0.6	6.3 ± 1.7
	GG	86.8 ± 57.2	6.0 ± 0.7	0.1 ± 0.1	0.5 ± 0.2	1.2 ± 1.0	8.9 ± 0.7	6.5 ± 1.7
	*P*-value	0.4383	0.3600	0.0764	0.9605	0.1729	0.6411	0.7895
	*P*_*adj*_-value	0.4109	0.3582	0.0532	0.9568	0.1556	0.6410	0.7623

^a^Urinary calcium-to-creatinine ratio (UCa/UCr). ^b^Urinary uric acid-to-creatinine ratio (UUA/UCr). *P* values which remain significant after performing Bonferroni correction (*p* ≤ 0.016) are shown in bold. The *P*_*adj*_ value was adjusted for gender and age.

**Table 6:** Association analysis between SNPs and subgroups of biochemical data in patients with kidney stone.

		Serum uric acid (%)		Serum calcium (%)		Serum creatinine (%)		UCa/UCr^a^ (%)	
	Genotype	>7.2 (mg/dl)	≤7.2 (mg/dl)	>10.2 (mg/dl)	≤10.2 (mg/dl)	>1.3 (mg/dl)	≤1.3 (mg/dl)	≥0.2	<0.2
*ALPLrs1256328*	TT	5 (4.2)	20 (7.3)	1 (11.1)	24 (6.4)	7 (8.6)	20 (6.2)	8 (9.4)	14 (5.1)
	CT	36 (30.5)	71 (25.9)	1 (11.1)	104 (27.7)	19 (23.5)	87 (27.0)	22 (25.9)	78 (28.4)
	CC	77 (65.3)	183 (66.8)	7 (77.8)	247 (65.9)	55 (67.9)	215 (66.8)	55 (64.7)	183 (66.5)
	*p* value		0.9030		0.7097		0.8432		0.4967
	OR (95% CI)		0.98 (0.67–1.42)		0.80 (0.25–2.59)		1.04 (0.69–1.58)		1.16 (0.76–1.75)
*CASRrs7627468*	GG	32 (27.6)	59 (22.3)	2 (22.2)	83 (22.7)	17 (21.8)	75 (23.9)	12 (14.3)	70 (26.0)
	AG	46 (39.7)	118 (44.5)	2 (22.2)	160 (43.9)	32 (41.0)	135 (43.0)	39 (46.4)	114 (42.4)
	AA	38 (32.7)	88 (33.2)	5 (55.6)	122 (33.4)	29 (37.2)	104 (33.1)	33 (39.3)	85 (31.6)
	*p* value		0.3227		0.2938		0.4194		0.0170
	OR (95% CI)		1.16 (0.86–1.57)		0.59 (0.23–1.57)		0.87 (0.62–1.22)		0.64 (0.44–0.92)
*RGS14rs12654812*	AA	12 (10.1)	25 (9.2)	1 (11.1)	36 (9.6)	8 (9.9)	29 (9.0)	10 (11.9)	21 (7.6)
	AG	44 (37.0)	100 (36.8)	3 (33.3)	138 (36.9)	26 (32.1)	122 (38.0)	32 (38.1)	99 (36.0)
	GG	63 (52.9)	147 (54.0)	5 (55.6)	200 (53.5)	47 (58.0)	170 (53.0)	42 (50.0)	155 (56.4)
	*p* value		0.7531		0.9895		0.7691		0.1854
	OR (95% CI)		1.06 (0.76–1.48)		1.01 (0.37–2.77)		0.94 (0.64–1.39)		1.30 (0.88–1.91)

The *p* value corresponding to the likelihood ratio test was obtained from a comparison with the null model and adjusted by gender and age.

^a^Urinary calcium-to-creatinine ratio (UCa/UCr). OR, odds ratio; CI, confidence interval. *P* values which remain significant after performing Bonferroni correction (*p* ≤ 0.016) are shown in bold.

### Lack of associations of genetic polymorphisms with the eGFR

According to previous studies, renal stones may cause renal impairment and decrease renal function. Therefore, we further calculated eGFRs based on the Modification of Diet in Renal Disease (MDRD) and Cockcroft-Gault (C-G) methods, widely used tools to predict renal function of nephrolithiasis patients. We tested the relationship between genetic polymorphisms and renal function. Among the SNPs we tested, no SNP was significantly associated with values of renal function calculated by either the MDRD or CG method (Supplementary Table S3).

### Effects of SNPs on gene expressions in different tissues

From data of the GTEx portal, we found that rs7627468 could influence the expression of the *IQCB1* (IQ motif containing B1) gene in the thyroid (*p* = 2.2 × 10^−7^) and the *EAF2* (ELL-associated factor 2) gene in the esophagus-mucosa (*p* = 4.1 × 10^−5^) (Table 7). We also found that rs12654812 has a cis-eQTL effect on *RGS14* (regulator of G-protein signaling 14) itself and is very likely to influence expressions of *MXD3* (MAX dimerization protein 3) in a variety of tissues. A significantly associated eQTL for rs12654812 was also identified for *FGFR4* (encoding fibroblast growth factor receptor 4) in the tibial nerve and *F12* (encoding coagulation factor XII) in esophagus mucosa. However, no significant eQTLs were found for rs1256328.

**Table 7:** Expression Quantitative Trail Loci (eQTL) results from Genotype-Tissue Expression (GTEx).

SNP ID	Gencode ID (ENSG00000-)	Gene Symbol	*p* value	Effect Size	Tissue	Actions
*CASR* rs7627468	173226.12	*IQCB1*	2.20E-07	−0.30	Thyroid	AA > AG > GG
	145088.4	*EAF2*	4.10E-05	−0.20	Esophagus-mucosa	AA > AG > GG
*RGS14* rs12654812	169220.13	*RGS14*	1.60E-44	−0.81	Cells-Transformed fibroblasts	GG > AG > AA
	169220.13	*RGS14*	2.50E-16	−0.71	Pituitary	GG > AG > AA
	169220.13	*RGS14*	3.10E-16	−0.26	Thyroid	GG > AG > AA
	169220.13	*RGS14*	1.60E-33	−0.66	Artery-aorta	GG > AG > AA
	169220.13	*RGS14*	4.60E-14	−0.49	Adrenal gland	GG > AG > AA
	169220.13	*RGS14*	1.70E-10	−0.58	Brain-cerebellum	GG > AG > AA
	213347.6	*MXD3*	1.0E-05	−0.22	Artery-aorta	GG > AG > AA
	213347.6	*MXD3*	2.40E-07	−0.18	Skin-sun exposed (lower leg)	GG > AG > AA
	213347.6	*MXD3*	9.60E-07	−0.20	Adipose-subcutaneous	GG > AG > AA
	160867.10	*FGFR4*	1.30E-11	−0.44	Nerve-tibial	GG > AG > AA

## Discussion

In this study, we showed that the TT genotype of rs1256328 in *ALPL* associated with higher susceptibility of nephrolithiasis in a Taiwanese population, which is consistent with a previous study in Icelandic population^25^. *ALPL* encodes tissue-nonspecific alkaline phosphatase (TNSALP), which is essential for the normal development of bones and teeth. TNSALP hydrolyses phosphate substrates such as pyrophosphate (PPi) and phosphorylated glycoproteins like osteopontin, and releases inorganic phosphate, thereby promoting appropriate calcification^26^. Animal study reported decreased bone mineralization in *ALPL*-knockout mice due to lower activity of phosphatase^27^. Furthermore, *ALPL* gene mutations were found in patients with hypophosphatasia, characterized by elevated urinary PPi excretion^28^. PPi was reported as a potent inhibitor of hydroxyapatite crystallization, which binds to the surface of basic calcium phosphate crystals and blocks subsequent crystal growth^29–31^. TNSALP is expressed in proximal tubules of the kidneys^28^ and frees phosphate through PPi hydrolysis^26^, which may further lead to kidney stone formation. Moreover, a previous study indicated associations of kidney stone diseases with other genes involved in phosphate regulation, including *VDR*, *klotho* (*KL*), and *sodium-hydrogen antiporter 3 regulator 1* (*NHERF1*)^12,32–35^, although rs1256328 (*ALPL*) had no significant eQTL effects on these genes. Since GTEx portal only collects limited cell types, and much evidence has suggested an important role of *ALPL* in the pathophysiology of nephrolithiasis via phosphate regulatory pathways. Therefore, further studies are required to elucidate the physiological effects of rs1256328 in human nephrolithiasis.

We observed that the AA genotype of rs12654812 in *RGS14* was significantly associated with nephrolithiasis susceptibility in a Taiwanese population, which is also consistent with a previous study on an Icelandic population^25^. Association of rs12654812 with nephrolithiasis was validated in a Japanese population^36^. However, studies in Chinese population were not totally consistence with each other^37,38^. *RGS14* encodes a member of the regulators of G-protein. Previous study on knockout mice indicated that RGS14 may regulate Ca^2+^ and affect downstream signals in neurons^39^. Similar cis-eQTL effects of rs12654812 on RGS14 expression were observed in nervous tissue in our study. However, this effect was not observed in kidney tissue. Since sample size of kidney tissue is limited by the current release of GTex, further studies are required to investigate the effects of rs12654812 on RGS14 expression in kidney tissue. Finally, we identified a significant eQTL in the tibial nerve for rs12654812 with *FGFR4* (Table 7), which was reported to be involved in renal phosphate homeostasis^40^. Consistent with previous studies, FGFR4 signaling is critical for the development of Calcium Nephrolithiasis.

RGS14 protein may restrict Ca^2+^ elevations^40^. The malfunctions of TNSALP, the encoding protein of *ALPL*, involve in the elevated urinary PPi excretion^28^. While AA genotype on rs12654812 leads to decreased expression of RGS14, Ca^2+^ level may subsequently increase. This concept is in consistent with the trend observed in our study (Table 5). It’s very likely that dysfunction of either ALPL or RGS14 influence the homeostasis of calcium and phosphate that increase the risk to nephrolithiasis. Therefore, further studies are needed to investigate whether rs1256328 (ALPL) and rs12654812 (RGS14) have synergic effects in the susceptibility of nephrolithiasis. Although we did not observe an association of rs7627468 in *CASR* with the susceptibility to nephrolithiasis, we found the rs7627468 correlate with the pH value and calcium/creatinine ratio of urine. CASR is a G-protein-coupled receptor located on plasma membranes. It senses and sets extracellular calcium ion levels by controlling renal calcium excretion and regulating parathyroid hormone secretion, thereby maintaining homeostasis of cellular calcium^41,42^. Moreover, several studies reported associations of *CASR* variations with nephrolithiasis or other kidney-related clinical manifestations. For example, two association studies on Italian populations indicated that the Arg990Gly (rs1042636) variant on *CASR* predisposes patients to primary hypercalciuria and nephrolithiasis^43,44^. Furthermore, Ala986Ser in exon 7 of *CASR* was repeatedly reported to be associated with a risk of kidney stones and serum calcium concentrations in different ancestries^45–47^. Our previous study also showed a significant association between *CASR* (rs17251221) and stone multiplicity in nephrolithiasis patients^13^. In addition, urinary acidification and urinary volume are two important factors associated with kidney stones^48^. For example, TRPV5^−/−^knockout mice demonstrated robust hypercalciuria and hyperphosphaturia without calcium-phosphate stone precipitation^49^. In the same study, researchers discovered that activation of the renal CASR can promote H^+^ excretion and downregulate aquaporin 2 leading to urinary acidification and polyuria^49^. These beneficial adaptations promoted the excretion of large amounts of soluble calcium, which was crucial to preventing the formation of kidney stones. This evidence indicates that CASR may be involved in kidney stone formation not only via controlling the calcium concentration, but also via regulating the urine pH. Furthermore, rs7627468 was associated with *IQCB1* expression in the thyroid, which encodes the nephrocystin protein that interacts with calmodulin^50^. rs7627468 was associated with *EAF2* expression in the esophageal mucosa. However, the physiological functions of *EAF2* in renal tissues are still unclear.

There are some limitations in this study. Data of the controls from the Taiwan Biobank were accessed through the Taiwan View website, which provides only summarized genotype counts, but not genotypes of individual subjects. For nephrolithiasis patients, important confounding factors, such as lifestyle, disease duration, and drug treatment, were unavailable, and we were therefore unable to adjust for the data. Furthermore, the modest sample size of nephrolithiasis patients collected in this study might have reduced the power of the statistical testing. Finally, although we observed eQTL effects of rs7627468 and rs12654812 *in silico*, further experiments are required to validate the effects of the SNPs on other genes and to elucidate the detailed subsequent mechanisms of the development of nephrolithiasis.

In summary, this study successfully replicated an association of SNPs in *ALPL* and *RGS14*, rs1256328 and rs12654812, with susceptibility to nephrolithiasis in a Taiwanese population. Our study further indicated that rs7627468 in *CASR* was associated with pH values and the calcium/creatinine ratio in urine of nephrolithiasis patients.

## Methods

### Longitudinal health insurance data from national health insurance research database

To investigate the prevalence of nephrolithiasis in Taiwan, we extracted calcium nephrolithiasis patients from the subset database (Longitudinal Health Insurance Database, LHID) of the National Health Insurance Research Database (NHIRD) using ICD-9-CM code 592.X. LHID database consists of medical claims data for 1,000,000 subjects randomly selected from the entire NHIRD (n = 23.5 million).

### Patient recruitment

Patients who fulfilled the diagnostic criteria for nephrolithiasis were recruited at Kaohsiung Medical University Hospital (KMUH). Our study conformed to the *Declaration of Helsinki*, and the design of the study and final report were performed with approval of the institutional review board of KMUH. We received informed consent from all subjects before any data were collected. Subjects were excluded if they had a history of renal tubular acidosis, renal failure, gout, primary or secondary hyperparathyroidism, chronic diarrhea, or cancer. In total, 454 calcium nephrolithiasis patients were diagnosed by a qualified urologist and were recruited. Each patient’s echographic and radiographic examinations of renal stones were retrieved along with clinical laboratory data, such as gender, age, and family history of nephrolithiasis. Blood and urine samples were collected at a random time point to measure total calcium, uric acid, and creatinine levels. All urinary parameters were corrected using urinary creatinine levels. Renal stone samples were obtained by either surgical intervention or spontaneous stone passage. Moreover, for the stone number analysis, patients with stone numbers exceeding one were allocated to the multiple-stone group, while patients with a single stone were grouped together. Patients without clear evidence for classifying them into either the multiple-stone or single-stone group were excluded from the stone number analysis.

The past medical history of the stone episode was retrospectively traced back as far as the patient’s medical record was available. Patients with only one episode were placed in the non-recurrent group; those with two or more symptomatic episodes within 6 months were allocated to the recurrent group. Since the medical history was retrospectively retrieved, patients without clear evidence for classifying them into either the single or recurrent group were excluded from the recurrence analysis.

### Genotypes of general population from Taiwan biobank

Taiwan Biobank is a prospective cohort of Taiwanese general population, in which cancer patients and subjects with nationalities other than Taiwan were excluded^51^. Among them, blood samples from 1517 subjects were sequenced. Four subjects exited the project afterwards, with 1513 subjects remaining. In this current study, SNPs frequencies for these 1513 subjects were obtained through the Taiwan View website (https://taiwanview.twbiobank.org.tw/index) as genotypic references of general population.

### DNA extraction

Peripheral venous blood was collected from patients and processed the same day. Blood was centrifuged at 3000 rpm for 10 min at 4 °C to separate serum and cells. The buffy coat containing peripheral blood mononuclear cells (PBMCs) was extracted and washed with red blood cell (RBC) lysis. Samples were mixed with a cell lysis buffer for several days after lysing the RBCs in lysis buffer. Proteins were precipitated using a protein precipitation solution. Finally, 95% isopropanol and 80% alcohol were used to isolate total genomic DNA.

### Candidate SNPs and genotyping

The SNPs investigated in this study were selected from a paper by Oddsson *et al*.^25^. Based on a GWAS, Oddsson *et al*. reported four SNPs associated with kidney stones in a European population. Among these SNPs, rs199565725 (*CLDN14*) was excluded from the study, because no frequency data were found in a search of the Taiwan View website. Furthermore, the minor allele frequency of this SNP and any other SNPs in high linkage (r^2^ ≧ 0.8) to rs199565725 are of low frequency (<0.05) in Asian population and Taiwanese population, according to HaploReg v4.1 and Taiwan Biobank, respectively. Therefore, three SNPs were analyzed in this study (Table 2). Genotyping was performed using the TaqMan Allelic Discrimination Assay (Applied Biosystems, Foster City, CA). A polymerase chain reaction (PCR) used a 96-well microplate with an ABI9700 Thermal Cycler (Applied Biosystems, Foster City, CA). After the PCR, fluorescence was measured and analyzed using System SDS software vers. 1.2.3 (Applied Biosystems).

### Statistical analysis

Statistical differences between patients and controls, multiple- and single-stone episodes, and between the recurrence and non-recurrence groups in genotype and allele frequencies were analyzed by a Chi-squared (χ^2^) test. Quantitative variables are expressed as the mean ± standard deviation (SD). A *t*-test or analysis of variance (ANOVA) was used to compare the means of continuous variables (clinical biochemical data and the eGFR) among different genotypes in nephrolithiasis patients. An analysis of covariance (ANCOVA) was applied to adjust for age and gender. The likelihood ratio test was used to analyze the association between biochemical data and each genotype, and each *p* value was adjusted for age and gender. Multiple testing was adjusted for SNP numbers using the Bonferroni correction. A significance level of 0.016 (0.05/3) was used. All statistical analyses were implemented using SAS 9.3 statistical software (SAS Institute, Cary, NC) for Windows. Regarding to Expression Quantitative Trait Locus (eQTL) analysis, we accessed the GTEx portal to investigate the effects of the SNPs on gene expressions in different tissues^52^.

## Supplementary information

Supplementary Information

## Data Availability

The datasets used and/or analysed in the study are available in the main text.
