# Supplementary material for: The rs1256328 (ALPL) and rs12654812 (RGS14) Polymorphisms are Associated with Susceptibility to Calcium Nephrolithiasis in a Taiwanese population
Source: Sci Rep. 2019 Nov 21;9:17296. doi: 10.1038/s41598-019-53261-8 (PMC6872875; doi:10.1038/s41598-019-53261-8)
Supplement: Supplementary file 1 — Supplementary Information [file 41598_2019_53261_MOESM1_ESM.pdf]

Supplementary Information for

The rs1256328 (*ALPL*) and rs12654812 (*RGS14*) Polymorphisms Are Associated with Susceptibility to Calcium Nephrolithiasis in a Taiwanese population

Wei-Chiao Chen[1,2+], Wan-Hsuan Chou[3+], Hou-Wei Chu[4], Chi-Chen Huang[5], Xiao Liu[6], Wei-Pin Chang[7*], Yii-Her Chou[2*], Wei-Chiao Chang[3,6,8*]

[1]Institute of Clinical Pharmacy and Pharmaceutical Sciences, College of Medicine, National Cheng Kung University, Tainan, Taiwan
[2]Department of Urology, Kaohsiung Medical University Hospital, Kaohsiung, Taiwan
[3]Department of Clinical Pharmacy, School of Pharmacy, Taipei Medical University, Taipei, Taiwan
[4]Institute of Biomedical Sciences, Academia Sinica, Taipei, Taiwan
[5]Graduate Institute of Neural Regenerative Medicine, College of Medical Science and Technology/Center for Neurotrauma and Neuroregeneration, Taipei Medical University, Taipei, 115, Taiwan
[6]Department of Medicine, The University of Chicago, Chicago, USA
[7]School of Health Care Administration, College of Management, Taipei Medical University, Taipei, Taiwan
[8]Master Program for Clinical Pharmacogenomics and Pharmacoproteomics, School of Pharmacy, Taipei Medical University, Taipei, Taiwan

[+] Wei-Chiao Chen and Wan-Hsuan Chou contributed equally to this work.
* Corresponding authors:
Wei-Chiao Chang (wcc@tmu.edu.tw); Yii-Her Chou (yihech@gap.kmu.edu.tw); Wei-Pin Chang (wpchang@tmu.edu.tw)

This file includes
Fig. S1 to S2
Table S1 to S3

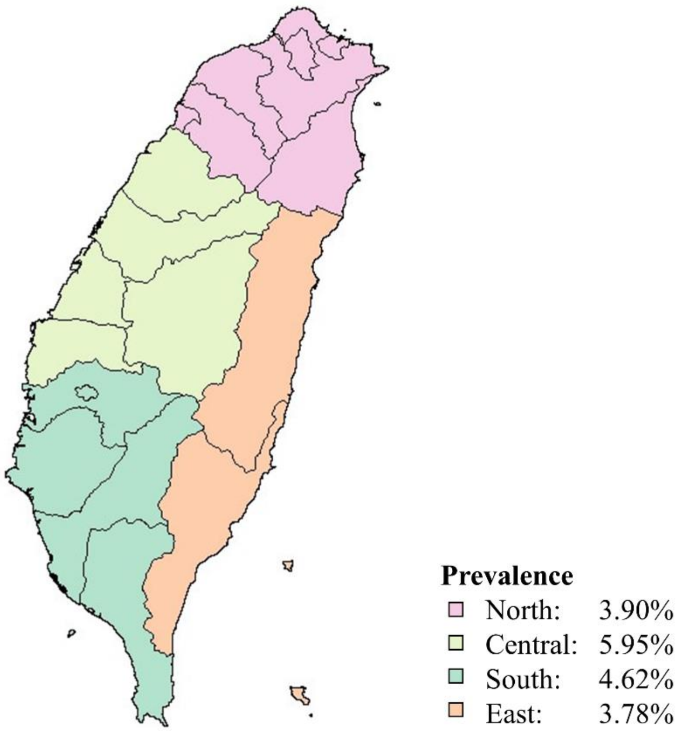

Fig. S1. Regional prevalence of nephrolithiasis in Taiwan

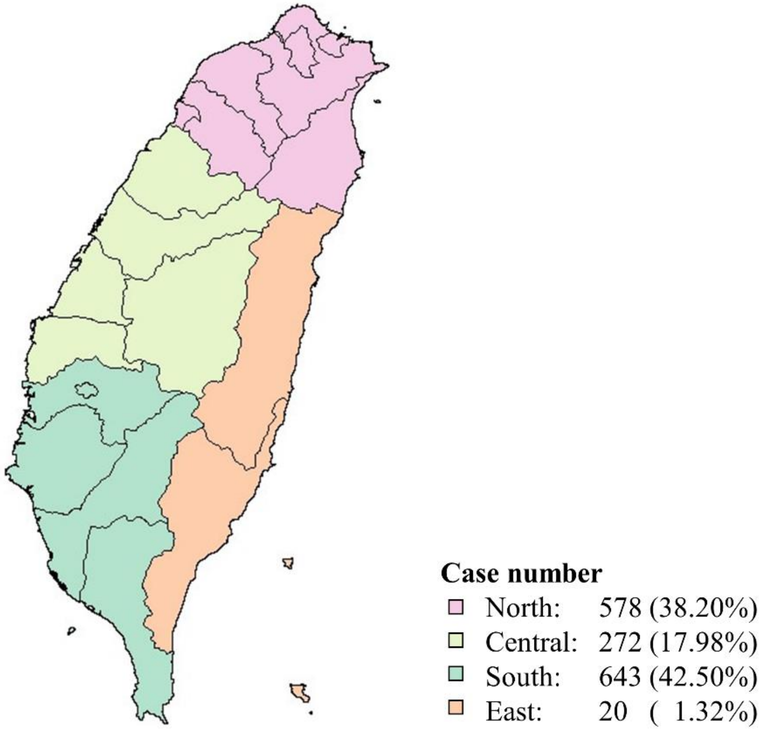

Fig. S2. Distribution of the subjects from Taiwan Biobank

Table S1. Comparisons of genotype and allele distributions between nephrolithiasis patients and controls.

SNP	Genotype	Genotype Frequencies		*P* value	Allele	Allele Frequencies		*P* value
		Case (%)	TWB[a] (%)			Case (%)	TWB[a] (%)	
ALPL	TT	33 (7.5)	58 (3.9)	**0.0005**	T	180 (20.5)	609 (20.2)	0.8313
rs1256328	CT	114 (26.0)	493 (32.7)		C	696 (79.5)	2403 (79.8)	
	CC	291 (66.5)	955 (63.4)					
CASR	GG	97 (22.8)	346 (23.2)	0.0613	G	379 (44.5)	1419 (47.5)	0.1170
rs7627468	AG	185 (43.4)	727 (48.7)		A	473 (55.5)	1567 (52.5)	
	AA	144 (33.8)	420 (28.1)					
RGS14	AA	39 (8.6)	68 (4.9)	**0.0072**	A	238 (27.2)	659 (23.7)	0.0346
rs12654812	AG	160 (37.1)	523 (37.6)		G	636 (72.8)	2121 (76.3)	
	GG	238 (54.3)	799 (57.5)					

[a]General population from Taiwan Biobank. *P* values which remain significant after performing Bonferroni correction ($p \leq 0.016$) are shown in bold.

Table S2. Association analysis between SNPs and stone frequency and stone numbers in patients with kidney stone.

Variant	Genotype	Multiple Stones (%)		*P*-value	Recurrence (%)		*P*-value
		Multiple	Single		Recurrence	Non-recurrence	
ALPL	TT	13 (6.1)	17 (8.6)	0.9533	13 (7.3)	14 (6.3)	0.7281
rs1256328	CT	63 (29.4)	47 (23.9)		44 (24.6)	64 (28.8)	
	CC	138 (64.5)	133 (67.5)		122 (68.1)	144 (64.9)	
CASR	GG	43 (20.6)	48 (25.3)	0.1050	40 (23.1)	47 (21.8)	0.9833
rs7627468	AG	89 (42.6)	83 (43.7)		73 (42.2)	96 (44.4)	
	AA	77 (36.8)	59 (31.0)		60 (34.7)	73 (33.8)	
RGS14	AA	24 (11.3)	12 (6.1)	0.1736	19 (10.6)	18 (8.1)	0.9405
rs12654812	AG	76 (35.7)	74 (37.6)		59 (33.0)	86 (38.9)	
	GG	113 (53.0)	111 (56.3)		101 (56.4)	117 (53.0)	

The *P* value was adjusted for sex and age. *P* values which remain significant after performing Bonferroni correction ($p \leq 0.016$) are shown in bold.

Table S3. Association analysis between SNPs and CGGFR/MDRD-S in patients with kidney stone

SNP	Genotype	MDRDs (ml/min/1.73 m^2)	*P*-value	CGGFR (ml/min)	*P*-value
ALPL	TT	71.92 ± 31.00	0.1704	82.23 ± 25.78	0.7298
rs1256328	CT	82.63 ± 27.10		87.28 ± 26.15	
	CC	80.21 ± 28.98		84.27 ± 30.39	
CASR	GG	82.87 ± 27.70	0.3126	84.92 ± 32.59	0.3628
rs7627468	AG	81.25 ± 27.32		88.37 ± 24.78	
	AA	77.49 ± 31.45		82.02 ± 30.86	
RGS14	AA	76.30 ± 29.42	0.6629	77.54 ± 21.87	0.1919
rs12654812	AG	80.03 ± 26.15		88.86 ± 28.03	
	GG	80.87 ± 30.27		83.58 ± 30.42	

The *P* value was adjusted for sex and age. *P* values which remain significant after performing Bonferroni correction ($p \leq 0.016$) are shown in bold.
